# 

**Published:** 1877-07

**Authors:** 


### Table of Contents.

### ORIGINAL COMMUNICATIONS.

A Case of Stricture in the Male Urethra of Twenty-Five Years' Standing. *By R. W. Westmoreland, M.D., Atlanta, Ga* ....... 193
The Narrow Meatus—How Produced. *By John Thad. Johnson, M.D., Atlanta, Ga* ........................................ 196
Mania a Potu. *By H. L. Wilson, M.D., Atlanta, Ga* ............... 198
Syphilis. *By Ferdinand King, M.D., Atlanta, Ga* ................. 200

### REPORTS OF SOCIETIES.

Atlanta Academy of Medicine ........................................ 208

### SELECTIONS.

An Expose of "Opium Antidotes." .................................. 216
A Case of Vesico Urethro-Vaginal Fistula, the Result of Venereal Ulceration ........................................ 218
Arkansas Hot Springs ............................................... 222
Fracture of the Patella ............................................ 223
A Case of Progressive Bulbar Paralysis ............................. 225
A Case of Glanders in the Human Subject ............................ 228
Recent Progress in Surgery ......................................... 230
Caseous Degeneration ............................................... 235

### EDITORIAL.

American Medical College Association ............................... 241
Convention of American Medical Editors ............................. 251
Editorial Correspondence ...........................................

### BIBLIOGRAPHICAL.

Micro-Photographs in Histology, Normal and Pathological ............ 255
Pamphlets ..........................................................

### EXCERPTA.

Sebaceous Tumors ................................................... 256
Remedy for Croup ...................................................
New Method of Treating Hemorrhoidal Tumors .........................
To Make Leeches Bite Promptly ......................................

